# Primary Cilia and Centrosomes in Neocortex Development

**DOI:** 10.3389/fnins.2021.755867

**Published:** 2021-10-21

**Authors:** Michaela Wilsch-Bräuninger, Wieland B. Huttner

**Affiliations:** Max-Planck-Institute of Molecular Cell Biology and Genetics, Dresden, Germany

**Keywords:** centrosome, cilia, neocortical development, neural progenitor, radial glia cells, spindle orientation, microcephaly

## Abstract

During mammalian brain development, neural stem and progenitor cells generate the neurons for the six-layered neocortex. The proliferative capacity of the different types of progenitor cells within the germinal zones of the developing neocortex is a major determinant for the number of neurons generated. Furthermore, the various modes of progenitor cell divisions, for which the orientation of the mitotic spindle of progenitor cells has a pivotal role, are a key parameter to ensure the appropriate size and proper cytoarchitecture of the neocortex. Here, we review the roles of primary cilia and centrosomes of progenitor cells in these processes during neocortical development. We specifically focus on the apical progenitor cells in the ventricular zone. In particular, we address the alternating, dual role of the mother centriole (i) as a component of one of the spindle poles during mitosis, and (ii) as the basal body of the primary cilium in interphase, which is pivotal for the fate of apical progenitor cells and their proliferative capacity. We also discuss the interactions of these organelles with the microtubule and actin cytoskeleton, and with junctional complexes. Centriolar appendages have a specific role in this interaction with the cell cortex and the plasma membrane. Another topic of this review is the specific molecular composition of the ciliary membrane and the membrane vesicle traffic to the primary cilium of apical progenitors, which underlie the ciliary signaling during neocortical development; this signaling itself, however, is not covered in depth here. We also discuss the recently emerging evidence regarding the composition and roles of primary cilia and centrosomes in basal progenitors, a class of progenitors thought to be of particular importance for neocortex expansion in development and evolution. While the tight interplay between primary cilia and centrosomes makes it difficult to allocate independent roles to either organelle, mutations in genes encoding ciliary and/or centrosome proteins indicate that both are necessary for the formation of a properly sized and functioning neocortex during development. Human neocortical malformations, like microcephaly, underpin the importance of primary cilia/centrosome-related processes in neocortical development and provide fundamental insight into the underlying mechanisms involved.

## Introduction

A characteristic feature of mammalian brains is the six-layered neocortex. Its size is a major determinant of the cognitive ability of a species (Molnar et al., 2019). The development of the neocortex to reach its appropriate size and proper cytoarchitecture is therefore of crucial importance. The proliferative capacity of neural stem and progenitor cells during embryonic/fetal development is a major determinant for the size of the mammalian neocortex (Lui et al., 2011; Florio and Huttner, 2014; Fernandez et al., 2016). Besides the number of progenitor cell divisions, the types of these divisions eventually determine how many neurons and glial cells are generated during cortical development. Alterations in the number and/or types of progenitor divisions can explain the differences in neocortex size observed between mammalian species, and aberrations in these parameters underlie certain human brain anomalies (Rakic et al., 2009; Florio and Huttner, 2014). Primary microcephaly is one of the most common abnormalities observed in human brain anatomy and has been shown to be caused by alterations in at least two dozen genes (Jayaraman et al., 2018; Heide and Huttner, 2021). Many of these genes code for centrosomal or ciliary proteins, showing the importance of these subcellular organelles for brain development.

Primary cilia are present on most, if not all, cells of the developing brain (as is the case for most other tissues). They are microtubule-based organelles originating at the basal body or mother centriole. The microtubules (MTs) are arranged in a ring of nine duplets (9 + 0 arrangement), the axoneme, which is surrounded by a specialized membrane (Satir et al., 2010). Primary cilia typically emerge from the cell surface and can receive and transduce signals from the environment. Intracellular transport in and out of this specialized organelle is tightly regulated by at least two distinct structures: the transition zone and ciliary necklace at the base of the axoneme on the one hand, and on the other hand the transition fibers or distal appendages, docking the basal body to the ciliary membrane (Graser et al., 2007; Tanos et al., 2013; Garcia-Gonzalo and Reiter, 2017). By separating the ciliary membrane domain from the bulk of the plasma membrane via the transition fibers and the transition zone, a special membrane composition of the cilium can be achieved (Mukhopadhyay et al., 2017; Reiter and Leroux, 2017; Garcia et al., 2018; Conduit and Vanhaesebroeck, 2020). Thus, the ciliary membrane is, for example, rich in signaling receptors which can mediate the specialized roles of primary cilia. The signaling pathway most commonly associated with ciliary function in brain development is Sonic hedgehog signaling (Huangfu et al., 2003; Saade et al., 2018; Andreu-Cervera et al., 2021). However, a variety of extracellular signals in addition to Sonic hedgehog have been shown, or are likely, to be received by the progenitor cells in the developing neocortex via their primary cilia (Park et al., 2019; Andreu-Cervera et al., 2021).

For signaling through ciliary receptors, transport into, and out of, the cilium has to take place. This transport is governed by multi-protein complexes, the intra-flagellar transport trains, which are driven by dynein and kinesin motors, transporting cargo along the axonemal MTs between the ciliary tip and the basal body (Mourao et al., 2016; Pigino, 2021). The basal body is a cylinder composed of 9 MT triplets and bears, besides the transitional fibers, a second set of appendages, the subdistal appendages, from which MTs reaching into the cytoplasm can originate (Sorokin, 1968; Kumar and Reiter, 2021). A constantly increasing set of proteins has been identified which localize to the different substructures of the cilium and basal body (Tanos et al., 2013; Kumar and Reiter, 2021; Tischer et al., 2021).

**FIGURE 1:**
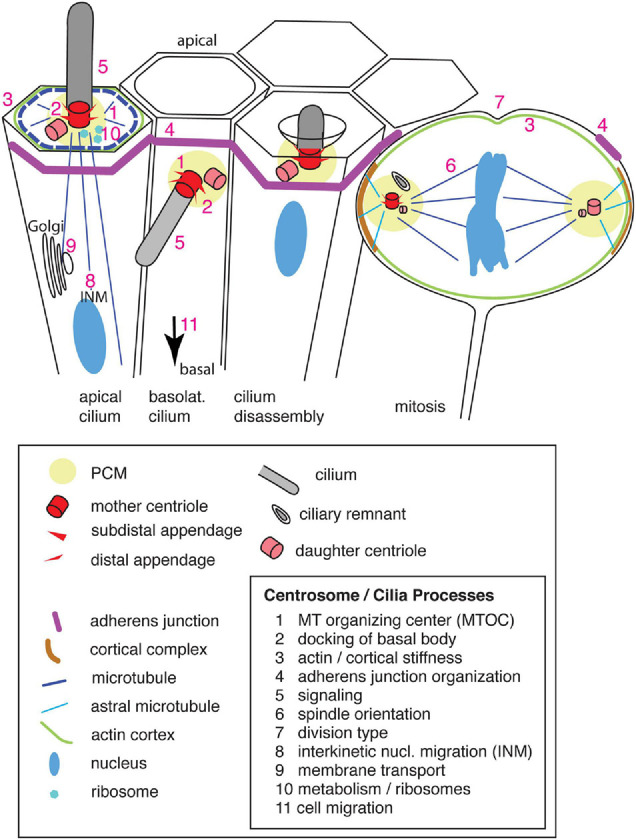
Primary cilia and centrosomes in the ventricular zone of the developing neocortex, their structure and components. Apical progenitors exhibit an apical primary cilium, which is connected by microtubules that originate from the basal body [microtubule organizing center (MTOC)], to the cortical actin and microtubule network and the adherens junction belt. The nucleus, connected by microtubules to the centrosome, undergoes interkinetic nuclear migration (INM). Vesicles are transported along microtubules from the Golgi complex toward the apical plasma membrane and primary cilium. Newborn basal progenitors exhibit a primary cilium on the basolateral plasma membrane, which initially is still integrated in the adherens junction belt prior to delamination (arrow). Primary cilia are disassembled prior to mitosis by resorption in a ciliary pocket. During mitosis the centrosomes act as spindle poles which are anchored via astral microtubules to the cell cortex (enriched for the NuMA/LGN/Gαi complex). A ciliary remnant is localized in the vicinity of the older mother centriole. Numbers indicate the sites of the corresponding processes. Only the apical domain of the cells is depicted.

The basal body of an interphase cell corresponds to the mother centriole, whereas the daughter centriole does not form appendages (Kumar and Reiter, 2021; Figure 1). Together with pericentriolar material (a specialized protein matrix) the two centrioles form the centrosome, the main MT organizing center (MTOC) of the cell. During G1/S phase of the cell cycle, the two centrioles separate and duplicate from their template a new set of (grand-)daughter centrioles (Nigg and Stearns, 2011; Kumar and Reiter, 2021). This duplication of the two centrioles underlies the generation of the second centrosome. During mitosis, the two centrosomes separate from one another and migrate apart to form the spindle poles (Figure 1). Each centrosome is subsequently distributed to one of the daughter cells. To allow mitosis, the primary cilium is resorbed by the cell in late G2/early prophase, and the basal body is freed to serve as part of one spindle pole. After mitosis, each of the two daughter cells can regrow a primary cilium.

In this review, we will focus on the cell biological processes which involve primary cilia and centrosomes during neocortical development. We will focus our attention on progenitor cells and will not address the role of centrosomes in neuronal migration. Since the signaling events related to ciliary sensing and to centrioles as signaling hubs in brain development have been extensively covered by other reviews (Park et al., 2019; Andreu-Cervera et al., 2021), we will focus on topics that complement these previously reviewed topics and which address the important roles of primary cilia and centrosomes in cortical progenitor biology. To this end, we will first briefly review the progenitor cell types in the developing neocortex.

## Progenitor Cell Types in the Developing Neocortex

During development, the neocortex originates from the neuroepithelium, a single layer of neuroepithelial cells. With the onset of neurogenesis, which takes place in mouse at around embryonic day 10 and in human at gestation week 10, the neuroepithelial cells transform into apical radial glia cells (aRG), which are more elongated and (like neuroepithelial cells) maintain a basal process contacting the pia throughout the cell cycle including during mitosis (Götz and Huttner, 2005; Taverna et al., 2014).

Divisions of progenitor cells in the developing neocortex after the onset of neurogenesis can occur either (i) in a proliferative manner, generating two daughter progenitors of the same type as the mother progenitor, (ii) in a self-renewing manner, generating a daughter progenitor plus either another type of progenitor or a post-mitotic neuron, or (iii) in a consumptive manner, generating two other types of progenitors or two neurons (Lui et al., 2011; Florio and Huttner, 2014; Taverna et al., 2014).

Progenitor divisions occur in the so-called germinal layers of the neocortex. Two such germinal layers can be distinguished: the ventricular zone (VZ), which is directly adjacent to the brain ventricles, and the sub-ventricular zone (SVZ), which is located basal to the VZ. Divisions of the stem and progenitor cells occur in either of these zones and, according to their location, apical progenitors (APs) and basal progenitors (BPs) can be distinguished. BPs are born from aRG mothers and can lose polarity, then giving rise to basal intermediate progenitors (bIPs). Some offspring of the aRG divisions maintain basal polarity (in particular during mitosis), then giving rise to basal radial glia cells (bRG) (Fietz et al., 2010; Hansen et al., 2010; Reillo et al., 2011). The latter progenitor type is thought to be particularly important for neocortex expansion in development and evolution (Lui et al., 2011; Florio and Huttner, 2014; Dehay et al., 2015). BPs are the major source of cortical neurons. The newborn neurons migrate further to the basal side of developing cortical wall, utilizing guidance cues from the progenitor fibers, and thereby form the multilayered cortical plate. The proper lineages from APs to BPs to neurons are not only of crucial importance for neocortex development and evolution, but also for preventing human brain malformations like microcephaly or heterotopias (Florio and Huttner, 2014; Wilsch-Bräuninger et al., 2016; Pinson et al., 2019; Thomas et al., 2019; Heide and Huttner, 2021; Liu et al., 2021).

When considering the role of the various progenitor cell types in the expansion of the neocortex during development and evolution, space constraints are of particular importance. One solution to avoid an overcrowding of mitoses at the ventricular surface due to an increase in the number of AP divisions is to shift progenitor mitoses to the SVZ as a secondary germinal zone. This shift becomes increasingly important with the temporal progression of neurogenesis and the evolutionary expansion of the neocortex (Fietz and Huttner, 2011; Florio and Huttner, 2014; Taverna et al., 2014). Another solution is the so-called interkinetic nuclear migration of APs, which we will discuss next.

## Interkinetic Nuclear Migration of Apical Progenitors

For cell biological reasons, notably the presence of an apical primary cilium, divisions of APs occur at the ventricular surface, a limited space. To this end, APs move their nucleus toward the ventricular surface for mitosis where they round up, while still keeping a slender connection to the basal lamina. After mitosis, the newborn daughter cells move their nuclei and cell bodies basally to yield more space at the ventricular side for other cells undergoing mitosis. This phenomenon of cells changing the position of their nuclei in concert with the cell cycle is called interkinetic nuclear migration (INM). INM has been shown to involve the apically directed MT motor dynein, which translocates the nucleus toward the ventricular surface, and actomyosin and the MT motor kinesin, which govern the basally directed nuclear movement (Sauer, 1935; Taverna and Huttner, 2010; Kosodo, 2012; Reiner et al., 2012; Miyata et al., 2014). Sun-Kash domain proteins tether the nucleus to the MT-based transport machinery (Zhang et al., 2009). These MTs originate from the centrosome, which in interphase is part of/associated with the primary cilium and thus docked to the apical plasma membrane of the AP (Kosodo, 2012; Figure 1). Thus, a crucial function of INM is to bring the nucleus (and hence the chromosomes) close to the centrosomes, which in APs are localized apically, reflecting the presence of an apical primary cilium. This close spatial proximity of the nucleus and the centrosomes then allows entry into mitosis, notably the formation of the mitotic spindle.

## Mitotic Spindle Orientation, Symmetric vs. Asymmetric Apical Progenitor Division, and Daughter Cell Fate

In contrast to divisions of BPs in the SVZ, which show a largely random orientation of the mitotic spindle (Fietz et al., 2010; Shitamukai et al., 2011), APs tightly control the orientation of their mitotic spindle and cleavage plane, which in the vast majority of cases is horizontal and vertical, respectively. This is of great importance for the fate of the daughter cells (Chenn and McConnell, 1995; Fish et al., 2006; Konno et al., 2008; Postiglione et al., 2011; Shitamukai et al., 2011). Given that APs exhibit intrinsic apical-basal cell polarity, symmetric proliferative AP divisions typically require a horizontal spindle orientation and vertical cleavage plane to ensure an equal distribution of the cellular components, notably the apical ones, to both daughter cells. In contrast, given that the apical domain of APs is relatively small, an oblique spindle orientation may result in the cleavage plane bypassing the apical domain and apical adherens junctional belt. This oblique cleavage plane orientation in turn would result in an asymmetric, self-renewing AP division where one of the daughter cells loses the contact to the ventricle and delaminates (Kosodo et al., 2004; Konno et al., 2008; Shitamukai et al., 2011).

Since one of the spindle poles contains the former basal body, whereas the other spindle pole is formed by the former daughter centriole, the two spindle poles are intrinsically asymmetric (Figure 1). The older mother centriole in one spindle pole largely maintains the appendages, whereas the younger mother centriole in the other spindle pole still needs to complete formation of the appendages (Breslow and Holland, 2019; Tischer et al., 2021) to be able to re-form a primary cilium. Moreover, a ciliary membrane remnant may be maintained at the older mother centriole and hence at that spindle pole/centrosome, which accelerates the re-formation of the primary cilium after mitosis (Paridaen et al., 2013). The former mother centriole is preferentially inherited by the daughter cell that retains stem cell properties (Wang et al., 2009; Paridaen et al., 2013). An asymmetry in centriole inheritance and cilium reformation after mitosis had been shown in cell culture cells to influence cell fate by a differential onset of Sonic hedgehog signaling and accumulation of PDGFRα (platelet-derived growth factor receptor α) on the reforming cilium in the two daughter cells (Anderson and Stearns, 2009). A modulator of the Notch signaling pathway, Mindbomb1 (Mib1), is asymmetrically associated with PCM-1–positive centriolar satellites in the chick neural tube. Intriguingly, the initial asymmetric distribution of Mib1 at the onset of mitosis is compensated in symmetric proliferative divisions by a Golgi complex-associated pool of Mib1, which is released during mitosis and can lead to an equal inheritance of Mib1 by the daughter cells (Tozer et al., 2017). A similar compensation mechanism by storage in organelles could exist for other asymmetrically localized centrosomal proteins and allow the switch between symmetric proliferative and asymmetric neurogenic divisions. Clearly, the initial asymmetry of the centrosomes contributes to the daughter cell fate. Another important determinant for daughter cell fate in the developing neocortex is the positioning of the centrosomes, i.e., the poles of the mitotic spindle of APs.

## Apical Progenitor Centrosome Positioning

**FIGURE 2:**
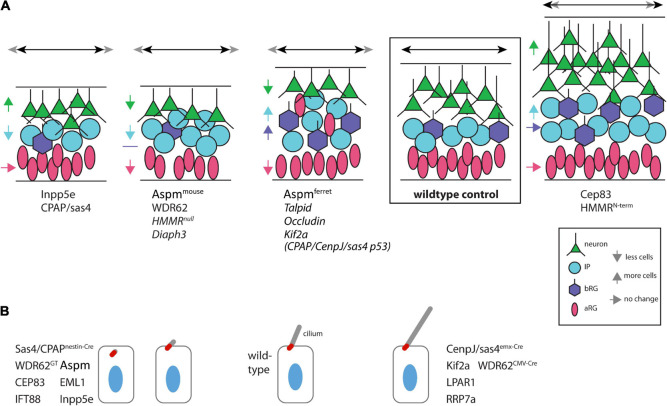
Range of phenotypes of mutations pertaining to primary cilia/centrosomes. **(A)** Schematic representation of the abundance of various cell types in the developing neocortex. The progenitor and neuron compositions of the developing neocortex upon mutation/knockdown of various genes are indicated. Names in italics indicate the lack of information on the abundance of bRGs in the mutant neocortex. Colored arrows indicate the change in abundance of the correspondingly colored cell type. Black double-sided arrows at the top indicate the changes in lateral expansion, with the width of the wildtype cortex (gray double-sided arrow) for comparison. Superscripts indicate species or mutation. Apical is down. **(B)** Primary cilium length. Schematic representation of the length of primary cilia (gray, basal body in red) of cells (nuclei in blue) in the developing neocortex as observed upon the indicated mutations. Superscripts indicate the conditional knockout conditions. Apical is up.

Astral spindle MTs are key players in the positioning of the centrosomes and hence the orientation of the mitotic spindle (Figure 1). Originating from the centrosome, they target the cell cortex by the Gαi/NuMA/LGN complex to generate a dynein-dependent pulling force for the correct spindle orientation (see di Pietro et al., 2016 for review). AuroraA and Polo-like kinase 1 (PLK1) are only two of the key players involved in mitotic spindle positioning that are located on the centrosome. Recently, hyaluronan-mediated mobility receptor (HMMR/RHAMM/CD168) was shown to support the action of PLK1 at the centrosome in the stabilization of astral MTs and, together with RanBP, to localize active Ran-GTP to the centrosome (Connell et al., 2017). Ran-GTP is required for the nucleation activity of the centrosome and the stability of MTs (Carazo-Salas et al., 2001). As a consequence, mitotic spindles of APs upon *HMMR* knockdown are less precisely horizontally positioned, less well centered within the mitotic AP, and exhibit shorter spindle MTs. This eventually results in a reduction of neural progenitor abundance. A surprisingly different outcome was reported when only the centrosome-localization domain of HMMR was deleted, resulting in an N-terminally truncated protein: AP spindle orientation was randomized, yielding more BPs and resulting in megalencephaly (Li et al., 2017). This phenotype is puzzling (and in contrast to the *HMMR* knockdown), as loss of function of other spindle-associated proteins is known to result in microcephaly (Figure 2A). Specifically, knockdown of *Aspm* in the ferret neocortex, which also results in an increase in BPs, causes microcephaly (Johnson et al., 2018; Figure 2A). A similar microcephalic phenotype has been observed in the case of mutations in human *ASPM* (Bond et al., 2003; Letard et al., 2018). Interestingly, infection of human iPSC-derived cerebral organoids with Zika virus (ZIKV), which causes congenital microcephaly in infected fetal human brains, results in a change in spindle orientation and premature neuronal differentiation of the infected cells (Gabriel et al., 2017; Saade et al., 2020). ZIKV (in particular the non-structural NS5 protein) affects the centriole structure. Upon infection, centrioles fail to accumulate appendage proteins and to form an elongated primary cilium (Gabriel et al., 2017; Saade et al., 2020).

As mentioned before, neurogenesis in the neocortex is accompanied by a switch from proliferative to self-renewing to consumptive divisions (Lui et al., 2011; Florio and Huttner, 2014; Taverna et al., 2014). Vargas-Hurtado et al. (2019) have recently shown that the stability of AP mitotic MTs (both astral and spindle MTs) in the embryonic mouse neocortex changes with the progression of neurogenesis: At a relatively early stage of neurogenesis (E13.5), when AP divisions are mostly asymmetric self-renewing, mitotic APs have thin MT bundles. In contrast, at late neurogenesis (E16.5), when symmetric proliferative divisions of APs are scarce and symmetric consumptive divisions prevail, MT bundles originating from the centrosome are thicker and more stable. Moreover, mitotic spindles at an early stage of neurogenesis contain more astral than spindle MTs. The early neurogenesis spindle can be mimicked at later stage by a partial depletion of the MT bundling factor Tpx2 (Vargas-Hurtado et al., 2019). The observation of a greater proportion of astral MTs at earlier stage of neurogenesis is consistent with the fact that the positioning of horizontal spindles for symmetric proliferative divisions needs to be precisely regulated to ensure the faithful symmetric distribution to the daughter cells of cell fate determinants in these divisions (Mora-Bermudez et al., 2014). This regulation is achieved by a sophisticated interplay between the NuMA/LGN/Gαi complex at the cell cortex and centrosomal factors like PLK1, AuroraA or ASPM (Morin and Bellaiche, 2011; Lancaster and Knoblich, 2012). Yet, additional players involved in AP centrosome positioning need to be discussed.

## Attachment of the Apical Progenitor Centrosomes to the Actin Cytoskeleton

Besides the classical centrosomal proteins which organize the mitotic spindle, like PLK1 and ASPM (Bond et al., 2002; Barr et al., 2004; Fish et al., 2006), some new players have recently appeared on stage which had not been known to be associated with the centrosome before. One example is the formin family protein diaphanous 3 (Diaph3), which had previously been shown to be involved in actin-driven processes, like the formation of the cleavage furrow and of filopodia (Bogdan et al., 2013). Now, Diaph3 was shown to localize to the centrosome, where it is involved in the assembly and stability of the mitotic spindle (Lau et al., 2021). Upon *Diaph3* knockdown, the orientation of the mitotic spindle is more randomized, and AP proliferation is reduced. These findings extend a previous study showing a role of Diaph3 in the regulation of the mitotic spindle checkpoint and reporting the occurrence of microcephaly upon Diaph3 depletion (Damiani et al., 2016).

An involvement of actin filaments in AP mitotic spindle formation and neurogenesis is in line with the results of neuron culture experiments implying that the centrosome not only organizes the MT asters, but also an actin network around the centrosome in newborn neurons in a PCM-1 (pericentriolar material-1 protein) -dependent manner (Meka et al., 2019).

Actin is enriched at the apical cell cortex of APs (Figure 1). It is part of the apical complex, a specific cytoskeleton-associated protein complex that includes Pals1/aPKCz. This apical complex is part of the apical cell cortex of epithelial cells and has a key role in epithelial polarity and junction assembly (Straight et al., 2004). The apical complex is of great importance for the proper formation of the neuroepithelium and of the neocortical cytoarchitecture in mouse embryos (Kim et al., 2010). Formation of the apical complex is supported by the centrosomal, mother centriole-associated proteins WDR62 and ASPM, which have a crucial role in regulating the number of progenitor divisions. *ASPM* and/or *WDR62* knockdown reduces the apical complex at the ventricular surface. This in turn appears to facilitate the delamination of progenitor cells, which then migrate to the SVZ (Jayaraman et al., 2016; Johnson et al., 2018). An important aspect of progenitor delamination pertains to the apical junctional complexes, the stability of which is influenced by the AP centrosomes, the next topic of our discussion.

## Role of Centrosome-Associated Proteins in the Interaction of the Cytoskeleton With Junctional Complexes

Delamination of progenitor cells is closely linked to the stability of the adherens junction belt lining the ventricular surface of APs (Taverna et al., 2014; Figure 1). The integrity of this highly dynamic zone of the developing neocortex, with its highly elongated APs that frequently divide, undergo INM, and give rise to cells that migrate basally, is ensured by adherens junctions and is of crucial importance for brain development (Veeraval et al., 2020). There are several lines of evidence that proteins localizing to the mother centriole influence the stability of the adherens junction complexes at the plasma membrane (see below). In the developing neocortical wall, adherens junctions are of critical importance for the distinction between progenitor types. APs are integrated into the apical adherens junction belt, whereas BPs typically leave the VZ after delamination from the apical adherens junction belt. These differences in adherens junction integration between the progenitor types has fundamental consequences for brain size (Rousso et al., 2012; Taverna et al., 2014; Stocker and Chenn, 2015; Wilsch-Bräuninger et al., 2016; Tavano et al., 2018).

One of the centrosome-associated proteins that influence adherens junction stability is AKNA (AT-hook transcription factor). It localizes to subdistal appendages on the mother centriole. Its expression is restricted to BP-genic APs and to BPs. AKNA can directly bind to MTs and is able to increase the MTOC activity at the centrosome and to influence MT stability independent of the centrosome. Besides modulating MTs, knockdown of *Akna* leads to stabilized junctions and blocks the actin re-modeling that normally occurs during delamination. Conversely, overexpression of *Akna* leads to a faster delamination by weakening junctional complexes, in particular junctional component CamSap3. Taken together, the apical constriction and delamination of radial glia cells, which is required for a timely progression of neurogenesis, involve AKNA (Camargo Ortega et al., 2019).

Another centriolar protein that affects MT stability and adherens junctions is Talpid3. It localizes to the distal end of centrioles (Yin et al., 2009; Kobayashi et al., 2014). Wang et al. (2020) have shown that Talpid3 together with Ninein localizes to mother centrioles and maintains adherens junction integrity by modulating MT stability. Knockdown of *Talpid3* (by either shRNA electroporation or conditional ablation) results (similar to *Akna* overexpression) in delamination and ectopic divisions of progenitor cells in more basal regions, leading to a reduced production of neurons (Wang et al., 2020; Figure 2A).

Together with AKNA and Talpid3, which influence adherens junctions by modulating MT stability, Cep83 is a third player in this class. Cep83 localizes to distal appendages of primary cilia (Joo et al., 2013; Tanos et al., 2013). When Cep83 is eliminated by a conditional knockout, the basal bodies dislocate from the apical plasma membrane without affecting centriole structure. Upon loss of Cep83, adherens junctions and the apical actin cortex of aRG are maintained, but increased in size (Shao et al., 2020). The enlarged apical endfeet of interphase APs show a mesh of fibrous MTs instead of the normal dense apical MT ring adjacent to the junctions. The resulting altered stiffness and contractile forces in the apical cortex upon the conditional *Cep83* knockout lead to a YAP-mediated proliferation phenotype and thereby to overproduction of neurons (megalencephaly) (Shao et al., 2020; Figure 2A).

In addition to these connections between centrosomal proteins and adherens junctions, a puzzling recent report has claimed that the tight junction protein Occludin is associated with centrosomes. Specifically, Bendriem et al. (2019) have reported that full-length Occludin is localized at both tight junctions and centrosomes of APs up to mid-neurogenesis, whereas a truncated form of Occludin localizes to the centrosome only, where it persists even after mid-neurogenesis when tight junctions have been dismantled. These claims are puzzling because even the truncated form of Occludin still contained one (rather than the usual four) transmembrane domain(s). Perhaps a more plausible explanation of the observations by Bendriem et al. (2019) is that the “centrosome” association of full-length and truncated Occludin reflected its localization in membranes structures in the immediate vicinity of the centrosome. Be that as it may, mice (or human cerebral organoids) lacking the full-length form of Occludin display a microcephaly phenotype caused by prolonged mitoses and premature neuron differentiation, which in turn involves a modulation of the spindle MTs and their cell cortex attachment via NuMA interaction (Figure 2A). A similar delay in mitotic entry and reduction of proliferation has previously been observed in transgenic MDCK cells expressing a mutated form of Occludin, where the authors also claimed centrosome association of Occludin (Runkle et al., 2011).

Having discussed the various cell biological features of the centrosomes in progenitor cells of the developing neocortex, we now turn to the interplay between the centrosomes and the primary cilium. Two prominent aspects of this interplay are (i) the docking of the basal body at the plasma membrane, and (ii) the disassembly of the primary cilium as a requirement to enter mitosis.

## Docking of the Basal Body at the Plasma Membrane

Since the centrosome serves a double role, contributing the basal body of the primary cilium during interphase and constituting a spindle pole during mitosis, the timing of the two functions has crucial consequences and must therefore be tightly regulated. Once the centrioles are engaged in a primary cilium, the cell is not able to divide—and while the old mother centriole-containing centrosome is acting as a mitotic spindle pole, no signaling can be received through the ciliary membrane. Interestingly, ablation of the axoneme/cilium in the telencephalon at mid-neurogenesis via conditional mutations in IFT proteins, does not result in significant changes in neocortex size (Snedeker et al., 2017; Shao et al., 2020). Importantly, these IFT mutations did not disturb the docking of the basal body at the plasma membrane. A different situation is observed when the docking of the mother centriole to the plasma membrane in interphase is impaired, as was shown to be the case upon disruption of the distal appendages in conditional *Cep83* knockout mouse embryos (Joo et al., 2013; Tanos et al., 2013). This knockout results in a strong increase in proliferation of radial glia cells and in neocortex size (Figure 2A). A possible explanation for the over-proliferation can be the before-mentioned altered apical MT network and a tension-induced increase in YAP signaling (Shao et al., 2020).

## Primary Cilium Disassembly: License for Mitosis

The switch from a plasma membrane-docked basal body to a spindle-pole (mother) centriole is a fundamental step in the cell cycle. Disassembly of the primary cilium prior to mitosis is thought to serve as a license for cell division (Sung and Li, 2011; Goto et al., 2013; Figure 1). Reduced anterograde IFT-transport into the primary cilium is a major mechanism for cilia resorption into the cell body (Liang et al., 2016; Sanchez and Dynlacht, 2016), although some membrane remnant may be preserved and remain as vesicle in the immediate vicinity of the centrosome (Paridaen et al., 2013). The MT-dependent resorption is regulated by AuroraA kinase: It phosphorylates histone deacetylase HDAF6 and thereby triggers tubulin de-acetylation in the axoneme (Pugacheva et al., 2007). Lysophosphatic acid (LPA), a phospholipid derivative, is involved in AuroraA-dependent primary cilium disassembly by activating the YAP/TAZ signaling pathway (Hu et al., 2021). In addition, phosphorylation of AuroraA is catalyzed by a calmodulin/calcium-dependent protein kinase. As a consequence, knockout embryos for the *LPA receptor1 (LPAR1)* show abnormally long primary cilia and reduced levels of both BPs and neurons (Hu et al., 2021; Figure 2B). A similar phenotype has been observed for mouse embryos mutant for either CenpJ/CPAP/Sas4, a MT-binding protein involved in centrosome maintenance and spindle orientation, or Kif2a, a kinesin motor protein (Figure 2B). Both these proteins colocalize at the basal body and are involved in ciliary disassembly (Broix et al., 2018; Ding et al., 2019). WDR62 can recruit both CenpJ/CPAP/Sas4 (together with Cep63 and ASPM) and Kif2a—via Cep170—to the basal body (Jayaraman et al., 2016; Zhang et al., 2019). Primary cilium disassembly is then triggered by the MT–de-polymerizing activity of Kif2a, or as a consequence of an altered structure (and function) of the transitional fibers on CenpJ/CPAP/Sas4–harboring mother centrioles. Hence, WDR62 is at the top of this cascade triggering ciliary resorption; however, conflicting findings regarding its effect on ciliary length have been reported: (i) Zhang et al. (2019) reported an increased length of primary cilia in WDR62 null mice and human cerebral organoids; in contrast, (ii) Jayaraman et al. (2016) show for *Wdr62* gene-trap mutant mice a reduction in the number (and size) of primary cilia; and (iii) Shohayeb et al. (2020), using CRISPR/Cas9-induced *Wdr62* mutations, which correspond to human microcephaly mutations, also show a reduction in number and size of primary cilia in the developing mouse neocortex (Figure 2B).

Not only centriolar factors like WDR62, ASPM, or CenpJ/CPAP can influence ciliary resorption: The phosphorylated form of the dynein light chain subunit DYNLT/Tctex-1, which is located at the transition zone, can also regulate ciliary disassembly (Li et al., 2011). In fact, the premature exit from the cell cycle and neuronal differentiation observed in cells in the neocortical wall upon shRNA-depletion of *DYNLT/Tctex-1* resemble the phenotype induced by *AuroraA*- or *HDAC6*-shRNA depletion.

A similar delay in cell cycle progression, together with impaired ciliary resorption, has recently been reported for a protein so far not known to be associated with primary cilia or centrosomes: Surprisingly, *ribosomal RNA processing 7 homolog A (RRP7A)* is a newly identified microcephaly gene. The RRP7A protein is localized to both nucleoli and primary cilia and, indeed, a delay in ciliary resorption has been observed in microcephaly patient-derived dermal fibroblasts. The mechanism for the delay in ciliary resorption upon RRP7A reduction, however, is not yet known. On the other hand, a function of RRP7A in RNA processing and ribosome biogenesis has been shown both in cell culture and in zebrafish embryos (Farooq et al., 2020). Which of these two—quite diverging—processes, ciliary resorption vs. ribosome biogenesis, is responsible for the microcephaly phenotype remains to be elucidated. The fact that phosphorylated S6 ribosomal protein, an effector of the primary cilia-related mTORC1 pathway, has been shown to localize around the mother centriole and at the apical side of mitotic aRG (Foerster et al., 2017) may speak in favor of a specific ribosome involvement at the centrosome to regulate cell proliferation and maybe primary cilium resorption.

The disassembly of the primary cilium as a license to enter mitosis is eventually followed by ciliogenesis in the daughter cells that emerge from mitosis. Two key aspects of ciliogenesis are biosynthetic membrane traffic and the composition of the ciliary membrane.

## Primary Cilium Formation and Vesicle Transport

Similar to ciliary resorption prior to mitosis, re-formation of a primary cilium from the centrosome/basal body after mitosis can regulate cell cycle progression (and thereby the speed of cell proliferation). Ciliogenesis occurs in several steps: After formation of the ciliary appendages, docking of Golgi-derived vesicles to the distal end of the mother centriole and the subsequent elongation of the MT-based axoneme and membrane addition take place (Sorokin, 1962; Pedersen et al., 2008; Kumar and Reiter, 2021). Proteins localizing to transitional fibers (distal appendages), like Cep164 and Ccdc41, recruit vesicles to the distal end of the mother centriole, likely in joint action with MTs and components of the vesicular transport machinery, like Rab8 (Nachury et al., 2007; Schmidt et al., 2012; Joo et al., 2013; Sillibourne et al., 2013; Kumar and Reiter, 2021). The MT-associated protein Eml1 may act in the same process: Lack of Eml1 in mouse mutant brains leads to shorter primary cilia, which are often located within a vesicle (Figure 2B). Interestingly, an abnormal structure of Golgi cisternae in the mutant cells, which likely will compromise apical transport of vesicles required for primary cilium formation, can explain this phenotype. The resulting mis-localization of progenitor cells in the mutant brains leads to heterotopias, which have been described for mouse mutants and for human patients (Uzquiano et al., 2019).

The transport of Golgi-derived vesicles presumably contributes, besides protein delivery, to the assembly of the primary cilium membrane. This membrane has a special protein and lipid composition that differs from that of the remaining plasma membrane (Conduit and Vanhaesebroeck, 2020). A specific set of signaling receptors and proteins required for signaling is enriched in this ciliary membrane. One of these is Inpp5e, a phosphatase controlling the phosphoinositide composition of the ciliary membrane and thereby its signaling activity (Bielas et al., 2009; Jacoby et al., 2009). Upon knockout of *Inpp5e* in mouse embryos, the primary cilia in the developing brain have an altered membrane structure and are shorter (Figure 2B). Interestingly, this results in a shift from indirect to direct neurogenesis, that is, with more neurons being born directly from aRG rather than via BPs. As this phenotype can be rescued by introducing the Gli3R repressor, this clearly shows that Inpp5e in the ciliary membrane is required for correct signal transduction (Hasenpusch-Theil et al., 2020).

Finally, the cell biological features of centrosomes and the primary cilium as well as their interplay discussed above lead to the crucial question of whether the distinct functions of these two organelles are necessarily linked to each other, or not.

## Primary Cilium or Centrosome? Who Takes the Lead?

With the plethora of phenotypes observed when manipulating centrosomes/primary cilia during neocortex development, there can be no doubt that these two organelles are of crucial importance for the correct formation of this brain structure (Thomas et al., 2019; Liu et al., 2021). However, the questions (i) whether it is merely the centrosome with its emanating cytoskeletal elements which is primarily responsible for the observed phenotypes (as suggested by Shao et al. (2020)), or (ii) whether this role should be attributed to the primary cilium, independent from the role of the basal body, remain unanswered. Certainly, the ciliary membrane is enriched in signaling receptors and is essential for transducing these signals to the cell body. Although the most prominent ciliary signaling pathway, that is, the Sonic hedgehog pathway, shows only low expression in the mouse dorsal telencephalon, a variety of other extracellular signal-induced pathways presumably operate via the primary cilia of the progenitor cells in the developing neocortex. Indeed, many ciliopathies (like Joubert syndrome) affect other brain regions (cerebellum) more than the forebrain. When the ciliary structure is impaired but still maintained in principle, only the initial establishment of the radial glia polarity, but not its maintenance, is affected, as was shown by Higginbotham et al. (2013) by conditional knockout of the ciliary membrane-associated small GTPase Arl13b. A similar result was obtained by Snedeker et al. (2017) when the authors conditionally deleted different axonemal components: Neither *IFT88* nor *Ttc21b (Ift139)* knockout in the telencephalon at mid-neurogenesis (using *emx1*-Cre) resulted in a significant phenotype. Even loss of centrioles (and primary cilia), upon defective centriole biogenesis, induced by a conditional knockout in *CenpJ/CPAP* combined with suppressed apoptosis (via *p53* deletion), leads to a cell cycle delay, altered spindle orientation and delamination of progenitors, but does not result in a change in neuron numbers (Insolera et al., 2014).

The ciliary membrane has been shown to be important for brain development at mid-neurogenesis: The ciliary membrane remnant that is inherited by one, but not the other, daughter of a mitotic aRG, has an influence on cell fate (Paridaen et al., 2013; Figure 1). The former daughter cell re-forms a primary cilium earlier after mitosis than the latter daughter cell who lacks the remnant. However, since the ciliary remnant is preferentially inherited by cells inheriting the mother centriole, it is difficult to distinguish whether the membrane or the centrosome composition makes the difference. Further support for the importance of the ciliary membrane comes from the finding that primary cilia occur on the basolateral, rather than apical, side of newborn BPs in the telencephalon (Wilsch-Bräuninger et al., 2012; Figure 1). In these basolateral cilia, the key structural components are not altered, but anchoring to the plasma membrane domain and the subsequent possibility to receive signals from the CSF or the intercellular space are different. As a consequence, cells bearing a basolateral cilium adopt a different fate than their sibling aRG and delaminate from the VZ. It is currently not possible to distinguish between the contribution of signaling molecules in the ciliary membrane or the transmission of these signals through the centrosome as signaling platform and origin of cellular and axonemal MTs. Further experimental evidence in favor of the involvement of the cilium independent of the centrosomal signaling hub is required.

## Future Aspects

In this review, we have focused on the cell biological features that characterize primary cilia and centrosomes, have outlined the interplay between these two organelles, and have discussed some functional aspects, however, without an in-depth discussion of the role of primary cilia in signaling. Of note, evidence for the importance of these organelles for cortical development has come from the dissection of certain human disorders (Romero et al., 2018; Thomas et al., 2019; Klingler et al., 2021). Many studies on the involvement of primary cilia/centrosomes in forebrain development have been triggered by clinical symptoms and human cortical malformations. The most prominent of these is congenital (primary) microcephaly, characterized by a reduced brain size at birth. At least half of the known microcephaly mutations (which comprise over 20 genes) are caused by centrosome- or primary cilia-related genes (Jayaraman et al., 2018; Gabriel et al., 2020). Other forebrain-affecting ciliopathies include Joubert syndrome and heterotopias (Romero et al., 2018; Klingler et al., 2021). Interestingly, mutations in some centrosomal/ciliary genes (e.g., *Cep83, IFT88*) can also lead to megalencephaly, i.e., larger brains (Li et al., 2017; Shao et al., 2020). However, an unexplained puzzle is why these mutations mostly affect cortical development although the expression of the genes concerned is not restricted to the cortex.

The large range of alterations in neocortex size upon mutations in ciliary/centrosomal genes reflects the differential involvement of the latter genes in cell division and fate determining processes, more specifically mitotic spindle orientation, interactions with the cytoskeleton, or establishment and stability of a primary cilium with signaling potential as described in the previous sections. The various actions of the products of ciliary/centrosomal genes have an impact on the differential production of cortical progenitor types, which exhibit various levels of proliferative potential. APs have provided a paradigm how the regulation of mitotic spindle orientation, together with the presence, stability and position of the primary cilium, determine the outcome of mitosis as being either proliferative, self-renewing or consumptive. A crucial topic of future research will be to investigate with similar depth which of these parameters also apply to the behavior of bRG, the BP type thought to have a key role in neocortex expansion, and to bIPs. It is worth noting that the mitoses in the SVZ of both of these BPs are less space-constrained than those of the APs from which these BPs originate.

In this context, it should be emphasized that the specific roles of the centrosomes and primary cilium in BPs have hardly been investigated so far. It can be expected that the input to the behavior of BPs through their ciliary membrane will be different from the signals in the cerebrospinal fluid received by the primary cilia on APs, in particular as many of the BP primary cilia are deeply embedded in a ciliary pocket. So far, almost no data on the molecular composition of the primary cilia nor of the centrosomes of BPs are available. The analysis of these primary cilia, part of which are often embedded in a ciliary pocket and which apparently exhibit a random position and orientation in the neocortical tissue, is much more challenging than that of the primary cilia at the ventricular surface. The diversity of the morphology of the cells in the SVZ, which comprise not only the different subtypes of bIPs and bRG, but also newborn neurons (Betizeau et al., 2013; Florio and Huttner, 2014; Kalebic et al., 2019), and which are densely packed, further increases the difficulty of studying their centrosomes/primary cilia. A start has been made by the observation that AKNA is specifically enriched in BPs compared to the other cortical cell types (Camargo Ortega et al., 2019). As BPs are the key players for cortical expansion during development and evolution, the contribution of their centrosomes and primary cilia to their proliferative potential can be expected to be a major trigger for neocortex size and to underlie certain human brain malformations. Now, with the possibility of targeted knockdown of proteins with CRISPR/Cas9 and a growing knowledge of cell type-specific transcriptomes, the door has been opened for a closer look at the difference between primary cilia and centrosomes among the various APs and BPs, hopefully resulting in further insight into the mechanisms underlying neocortex expansion.

## Author Contributions

MW-B designed the figures. MW-B and WH wrote the manuscript. Both authors contributed to the article and approved the submitted version.

## Conflict of Interest

The authors declare that the research was conducted in the absence of any commercial or financial relationships that could be construed as a potential conflict of interest.

## Publisher’s Note

All claims expressed in this article are solely those of the authors and do not necessarily represent those of their affiliated organizations, or those of the publisher, the editors and the reviewers. Any product that may be evaluated in this article, or claim that may be made by its manufacturer, is not guaranteed or endorsed by the publisher.
